# Supplementary material for: Collecting mortality data via mobile phone surveys: A non-inferiority randomized trial in Malawi
Source: PLOS Glob Public Health. 2022 Aug 11;2(8):e0000852. doi: 10.1371/journal.pgph.0000852 (PMC10021539; doi:10.1371/journal.pgph.0000852)
Supplement: S1 Fig — Notes: Respondents were first asked if any of the interview questions made them upset, and if so, they were asked to indicate the severity of their negative feelings. In the group asked mortality-related questions, the strength of negative feelings was associated with recent reports of deaths (p = 0.02). In the mortality-related group group, the width of each bar is proportional to the number of respondents reporting different numbers of deaths during the interview. (PDF) [file pgph.0000852.s002.pdf]

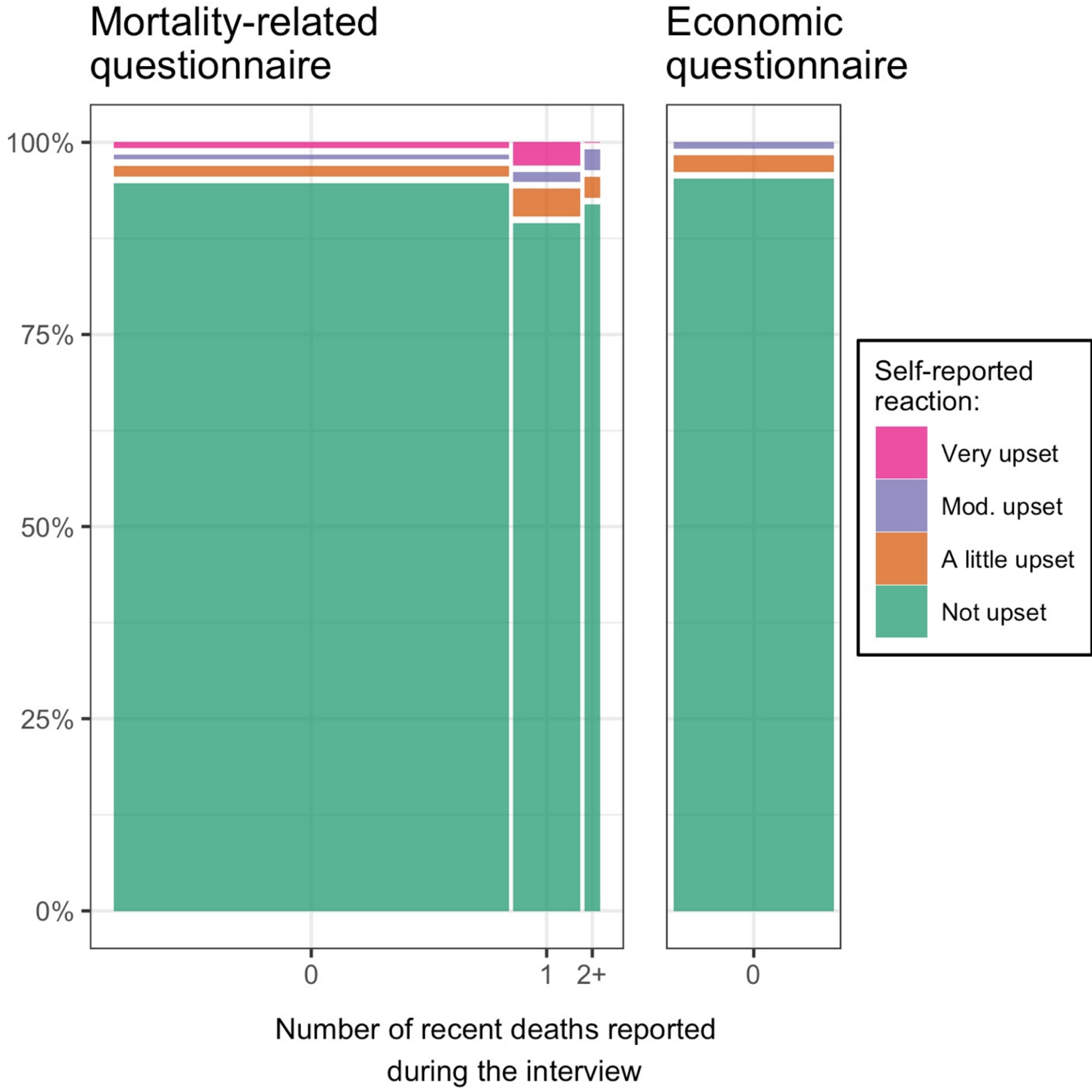

Mortality-related questionnaire
Economic questionnaire
100%
75%
50%
25%
0%
0
1
2+
0
Number of recent deaths reported during the interview
Self-reported reaction:
Very upset
Mod. upset
A little upset
Not upset
