# Supplementary figures and images for: Collecting mortality data via mobile phone surveys: A non-inferiority randomized trial in Malawi
Source: PLOS Glob Public Health. 2022 Aug 11;2(8):e0000852. doi: 10.1371/journal.pgph.0000852 (PMC10021539; doi:10.1371/journal.pgph.0000852)

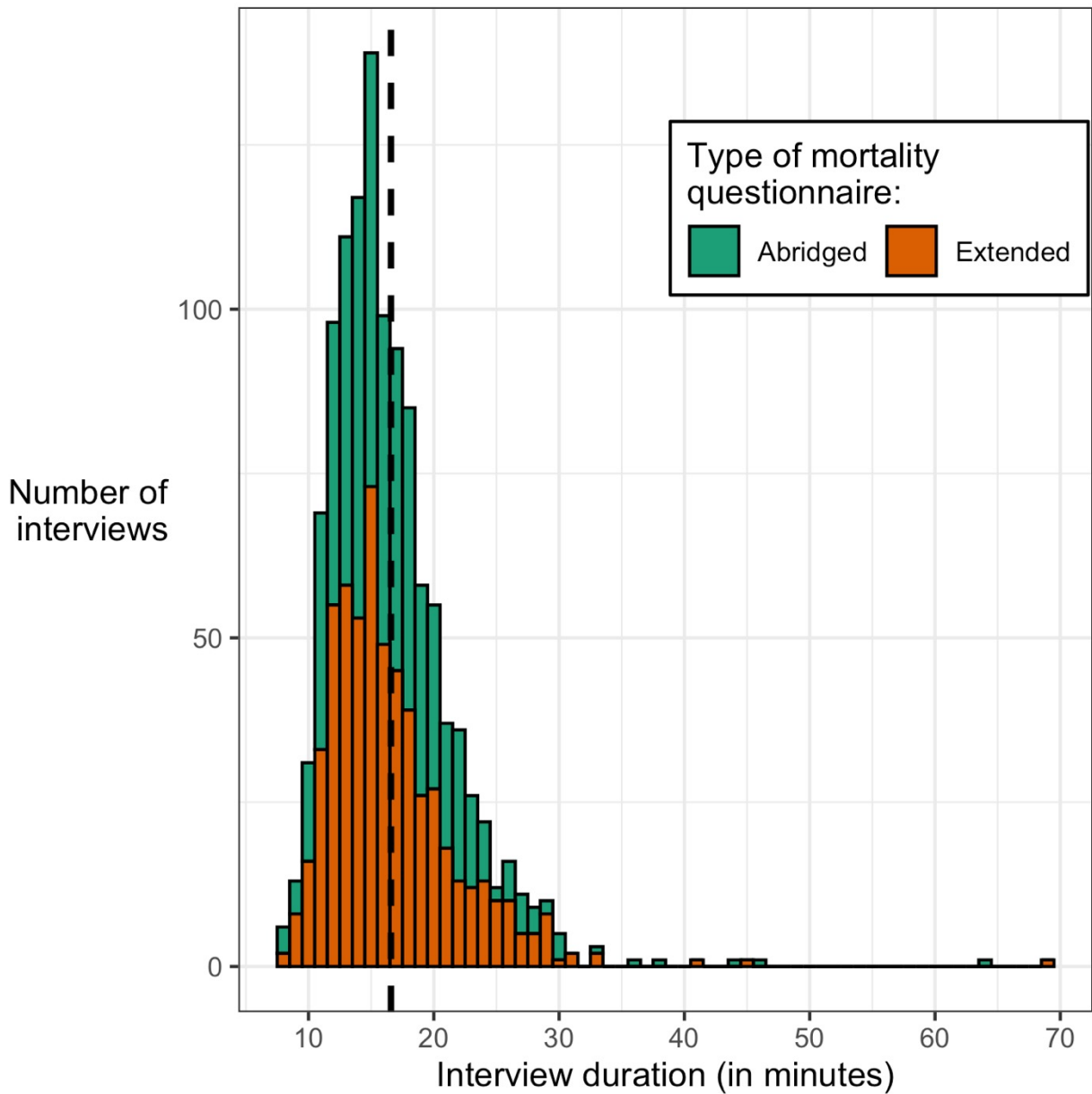

Supplement: S2 Fig — Notes: The dashed vertical line represent the mean duration of interviews in the group asked questions related to mortality, regardless of questionnaire type (abridged vs. extended). (PDF) [file pgph.0000852.s003.pdf]
