# Supplementary material for: Collecting mortality data via mobile phone surveys: A non-inferiority randomized trial in Malawi
Source: PLOS Glob Public Health. 2022 Aug 11;2(8):e0000852. doi: 10.1371/journal.pgph.0000852 (PMC10021539; doi:10.1371/journal.pgph.0000852)
Supplement: S1 Table — Notes: “Busy/call-back” refers to respondents who consented to being interviewed, indicated that they would prefer being called-back at a later time, and could not be reached again before the completion of the study. (DOCX) [file pgph.0000852.s004.docx]

|  | **Mortality-related**  **questionnaire (N=1270)** | **Economic**  **questionnaire (N=413)** | **Overall (N=1683)** |
| --- | --- | --- | --- |
| **Study result** |  |  |  |
| Completed interview | 1174 (92.4%) | 378 (91.5%) | 1552 (92.2%) |
| Refusal | 57 (4.5%) | 21 (5.1%) | 78 (4.6%) |
| Busy/call-back | 24 (1.9%) | 8 (1.9%) | 32 (1.9%) |
| Interview discontinued | 15 (1.2%) | 6 (1.5%) | 21 (1.2%) |
