# Supplementary material for: Collecting mortality data via mobile phone surveys: A non-inferiority randomized trial in Malawi
Source: PLOS Glob Public Health. 2022 Aug 11;2(8):e0000852. doi: 10.1371/journal.pgph.0000852 (PMC10021539; doi:10.1371/journal.pgph.0000852)
Supplement: S2 Table — Notes: Supervisor follow-ups were conducted 1–2 days after the initial interview. The supervisors were not aware of the answers provided about self-reported feelings during the initial interview; nor were they aware of the respondents’ assignment to the different study groups. (DOCX) [file pgph.0000852.s005.docx]

|  |  | **Interviewer data** | |
| --- | --- | --- | --- |
|  |  | No negative feelings | Some negative feelings |
| **Supervisor follow-up** | **Mortality questionnaire** |  |  |
|  | No negative feelings | 57 (91.9) | 4 (22.2) |
|  | Some negative feelings | 5 (8.1) | 14 (78.8) |
|  | Total | 62 (100.0) | 18 (100.0) |
|  | **Economic questionnaire** |  |  |
|  | No negative feelings | 15 (88.2) | 0 (0.0) |
|  | Some negative feelings | 2 (11.8) | 7 (100.0) |
|  | Total | 17 (100.0) | 7 (100.0) |
