# Supplementary material for: Collecting mortality data via mobile phone surveys: A non-inferiority randomized trial in Malawi
Source: PLOS Glob Public Health. 2022 Aug 11;2(8):e0000852. doi: 10.1371/journal.pgph.0000852 (PMC10021539; doi:10.1371/journal.pgph.0000852)
Supplement: Attachment — Submitted filename: reply-to-reviewers-07022022sh.docx [file pgph.0000852.s006.docx]

**Reviewer #1:**

**The authors report a randomized trial to assess the acceptability of a mobile phone survey to enhance real-time mortality surveillance in Malawi. This is noble and important work and I applaud the authors for pursuing this line of investigation. The paper is clearly written, and the findings are accurately described and contextualized. I think this article will be of interest to public health officials and NGOs who collaborate in similar settings.**

*We thank the reviewer for this kind assessment of our work.*

**Despite the article’s strengths, there are several weaknesses that I think could be addressed to strengthen the manuscript. My biggest question is why a survey about economics in a low-income country was determined to be an appropriate comparator for acceptability of a mobile phone survey. Would the authors imagine that asking about finances during a global pandemic with economic repercussions in a low-income country may be perceived as intrusive in any way? Yes, the authors mention that this economic survey is “highly acceptable”, but I think it is worth considering and discussing a bit more. I do not think this takes away from the value added of this study, but I do think it is worth considering.**

*We have selected this topic as a “control” topic due to its frequent inclusion in mobile phone surveys conducted since the beginning of the pandemic. As such, it represents a good benchmark against which to evaluate the acceptability and feasibility of collecting mortality data via mobile phone surveys. The reviewer is correct however that asking questions about recent economic activity might nonetheless generate negative reactions, in particular during a pandemic or another kind of economic crisis. We thus clarified in our text that this “control” topic might still elicit a number of negative reactions, but that this level of reactions is generally deemed acceptable as demonstrated by the frequent inclusion of the topic in mobile phone surveys. This description is included in the introduction, and reads as follows:*

*“In a randomized trial conducted among mobile phone users in Malawi, we assessed whether participation and emotional responses differed between mobile users who were asked to complete a mortality-related interview and users asked to complete an interview about their recent economic activity. While this “control” topic might generate negative reactions from respondents, particularly at times of increased economic stress, it is widely perceived as acceptable to investigate via MPS, and has repeatedly been included in MPS conducted since the beginning of the COVID-19 pandemic. It thus represents a useful benchmark against which to evaluate the feasibility of mortality-related MPS. Our results provide evidence that mortality data collection via MPS is acceptable. They suggest that MPS might constitute a useful tool for strengthening the surveillance of mortality during epidemics and other crises affecting population health in countries with deficient civil registration systems.” (p. 6)*

**Also, can the authors provide any estimates on phone ownership in Malawi? This may help contextualize the limitation of this method of surveillance being limited to phone owners. I would imagine that families that do not own phones may be more economically disadvantaged and perhaps have higher mortality rates. Otherwise, I have minor comments below for the authors to consider.**

*This is an excellent suggestion. We have provided AFROBAROMETER data on this issue in the new figure 1, and we discuss access to mobile phones in our presentation of the study context. This is the most recent nationally-representative on this topic currently available. We have also included comparisons with estimates of access to mobile phones in neighboring countries. This now appears in the methods section as follows:*

*“The coverage of mobile phone networks is extensive in Malawi: in the nationally-representative Afrobarometer survey conducted in November-December 2020, mobile phone service was available in all randomly selected communities located in urban areas, and in 95% of communities located in semi-urban or rural areas. At that time, 56% of all adults in Malawi personally owned a mobile phone, up from 48% in 2014/15. Ownership of mobile phones however remains more limited in Malawi than in other eastern and southern African countries. According to Afrobarometer data, for example, 62% of adults in Mozambique, 75% of adults in Tanzania and more than 90% of adults in countries like Kenya, Botswana or Eswatini personally owned a mobile phone in 2020/21. There are also large disparities in access to mobile phones between socio-economic groups in Malawi. In urban areas, more than 90% of adult men, and close to 75% of adult women reported that they owned a mobile phone (Figure 1). In semi-urban and rural areas, these proportions were 63% and 42%, respectively. Since the beginning of the COVID-19 pandemic, several MPS have been conducted in Malawi [30–32], but they have not reported recent mortality trends. (pp. 7-8)”*

**Abstract:
-Perhaps it would help the reader understand the primary outcome better if “cooperation rate” were defined in the abstract. This is not a commonly used or widely understood term. Just this just mean that respondents filled it out?**

*Thank you for this suggestion, we have now provided a definition of “cooperation rate” in our abstract.*

**-I think it would help the reader understand the size of the study by including the number of phone numbers that were sent the survey and how many respondents there were in the Abstract.**

*We have also added this information to the abstract.*

**Introduction:
-Minor, but line 58 should be “and” not “an”.**

*This has been corrected.*

**-In general, this reads quite long and may benefit from some tightening up of the language, though the content is good and sets the stage for the study well. Much of the content can be moved to the Discussion, in my opinion.**

*Thank you for this suggestion. We have shortened the introduction, primarily by moving the description of various methods of mortality surveillance to the discussion section of our papers. We agree that this makes the manuscript clearer.*

**-Minor, but please be consistent with the use of COVID-19. Currently there are some areas where it reads COVID and others that read COVID-19.**

*We have revised this, using “COVID-19” throughout the text.*

**-The final paragraph, which is currently a sentence, does not clearly state the rationale or potential implications of this line of work. Though this is stylistic, I think this would help the authors succinctly frame the need and the potential implication of this work in a paragraph that many readers look for this information.**

*We have reformulated this paragraph, to highlight our main contributions. This paragraph now reads as follows:*

*“In a randomized trial conducted among mobile phone users in Malawi, we assessed whether participation and emotional responses differed between mobile users who were asked to complete a mortality-related interview and users asked to complete an interview about their recent economic activity. While this “control” topic might generate negative reactions from respondents, particularly at times of increased economic stress, it is widely perceived as acceptable to investigate via MPS, and has repeatedly been included in MPS conducted since the beginning of the COVID-19 pandemic. It thus represents a useful benchmark against which to evaluate the feasibility of mortality-related MPS. Our results provide evidence that mortality data collection via MPS is acceptable. They suggest that MPS might constitute a useful tool for strengthening the surveillance of mortality during epidemics and other crises affecting population health in countries with deficient civil registration systems.” (p. 6)*

**Methods:
-In the Randomization section it is unclear if the interviewers knew which group the potential participant would potentially pertain to at the time of the phone call. Was the randomization done prior to the phone call? During the phone call? This could be clearer.**

*We have clarified this important aspect. Interviewers remained blind to the respondent’s assignment until after they had confirmed his/her eligibility for the study. This now reads as follows:*

*“Randomization: after interviewers successfully contacted a mobile phone number, they introduced themselves to the user and stated that they were conducting a survey about the impact of the COVID-19 pandemic on several aspects of the lives of Malawians. They asked the phone user if they were interested in learning more about the study. If so, they assessed the user’s eligibility by asking 4 questions about gender, age and residence. Mobile users who did not meet the age criterion and those whose sampling stratum was already filled were told that they were not eligible for the study. When initially placing a call to a selected mobile number, study interviewers were unaware of the mobile user’s assignment to the mortality or economic questionnaire. They remained unaware of that assignment until the user’s sampling stratum had been determined and eligibility had been confirmed.” (p. 9)*

**Results:
-Table 1: I’m not sure “treatment” is the appropriate word since this is a survey. Perhaps clarifying the language by stating “Mortality Survey Respondents” or something to that effect would be more appropriate and “Economic Survey Respondents” instead of Control.**

*This is a very useful suggestion. We have adopted economic vs. mortality questionnaires in the course of our paper, we have also adopted this designation in our figures and tables.*

**Reviewer #2: Reviewer comments**

**PGPH-D-22-00492 - Collecting mortality data via mobile phone surveys: a non-inferiority randomized trial in Malawi


Thank you for providing me an opportunity to review your research. This is a welcome addition to the field of evidence for the use of Mobile phones for quick surveys of mortality.

I must congratulate on getting out a fairly well written without grammatical errors or spelling mistakes. I have provided some comments within the PDF attached - some of them are suggestions or observations on your text with the hope that you may modify or provide justifications for not doing so.**

*Thank you for this kind assessment of our work.*

**One key point was about the sample size calculation section. It is not clear what is the response level expected of the treatment arm and I was unable to replicate your sample size calculations. (see PDF)**

*We have clarified our hypotheses in the revised version of our paper. Specifically, we set p1 and p2 at 0.86, then set a 0.05 non-inferiority margin. The revised section of our work reads as follows:*

*“Sample size: We used standard formulae for the determination of sample size in non-inferiority trials [36]. Based on preliminary pilot data, we assumed a cooperation rate of 86 per cent for both the mortality and economic questionnaires, and we set a non-inferiority margin of 5 percentage points. Our null hypothesis was that the cooperation rate among mobile users asked to complete the mortality questionnaire is lower than among those allocated to the economic questionnaire by more than 5 percentage points. Our alternative hypothesis was that the cooperation rate among those asked to complete the mortality questionnaire is lower by less than 5 percentage points. To test this hypothesis with a 3:1 allocation ration, 80% power and alpha = 0.05, we required at least 1,194 mobile subscribers allocated to the mortality questionnaire and 398 mobile subscribers allocated to the economic questionnaire. Sample size calculations were performed using the SampleSize4ClinicalTrials package in R.” (pp.10-11)*

*The code to reproduce our calculations in R is as follows:*

ssc_propcomp(design = 3L, ratio = 3, alpha = 0.05, power = 0.8, p1 = 0.86, p2=0.86, delta = 0.05).

*We also clarify questions raised in the annotated pdf:*

- *Our definition of cities matches the definition of “urban” used by the Malawi NSO. As such the % in cities is directly comparable to the proportion urban reported in the census, for example. Other areas are “rural”, according to the NSO. We added a discussion of urban bias in our paragraphs on “limitations”.*
- *We conclude that feelings are transitory based on our assessment of respondents’ feelings a) at the end of the interview, and b) during supervisors’ follow-up calls. We ask respondents if they experienced any negative reactions during the course of the interview, and if so, we ask again at the time of the end of interview and during follow-up whether they are “ok now”. The overwhelming majority of respondents indicated being ok, which informs our assessments that negative feelings are transitory. The follow-up calls by the clinical psychologist to the respondents who reported not being ok lends further credence to this interpretation. We clarify this in the text on page 20.*
